# Urinothorax Secondary to Obstructive Uropathy: A Rare Cause of Pleural Effusion

**DOI:** 10.7759/cureus.108274

**Published:** 2026-05-04

**Authors:** Ivy Paul, Ajeesh K Padmanabhan, Dhanasekar T, Hari Prasad

**Affiliations:** 1 Respiratory Medicine, Sri Ramachandra Institute of Higher Education and Research, Chennai, IND

**Keywords:** exudative pleural effusion, hydronephrosis, hydroureteronephrosis, nephrolithiasis, obstructive uropathy, percutaneous nephrolithotomy (pcnl), pleural effusion, pleural fluid to creatinine ratio, renal calculus, urinothorax

## Abstract

Urinothorax is an exceptionally rare cause of pleural effusion characterized by the abnormal accumulation of urine in the pleural space, typically secondary to genitourinary trauma or obstructive uropathy. We report the case of a 54-year-old male who presented with acute right-sided chest pain, colicky abdominal pain, and rapidly worsening respiratory distress. Serial imaging demonstrated a rapidly refilling massive loculated pleural effusion alongside a 3.3 cm right pelviureteric junction calculus, causing moderate hydroureteronephrosis. Ultrasound-guided thoracentesis yielded an exudative fluid with a distinct uriniferous odor. Simultaneous pleural fluid and serum creatinine levels were 0.98 mg/dL and 0.8 mg/dL, respectively, yielding a diagnostic ratio of 1.22. Despite the absence of definitive radiological evidence of a retroperitoneal urinoma or urinary extravasation, the diagnosis of urinothorax was confirmed. The patient achieved complete resolution following repeated therapeutic aspirations, right percutaneous nephrolithotomy, and double-J stenting. This case underscores that urinothorax can present with an atypical exudative profile and without classic radiological findings. Clinicians must maintain a high index of suspicion in patients presenting with rapidly accumulating pleural effusions and concurrent obstructive uropathy, relying on the pleural fluid-to-serum creatinine ratio for definitive diagnosis.

## Introduction

Urinothorax is defined as the abnormal accumulation of urine within the pleural cavity and represents one of the rarest etiologies of pleural effusion [1,2]. The condition predominantly arises from two distinct mechanisms, namely, traumatic injury to the genitourinary tract (often iatrogenic) or obstructive uropathy [1]. The pathophysiology involves retroperitoneal extravasation of urine, which subsequently migrates into the pleural space via diaphragmatic lymphatics or through direct anatomical defects in the diaphragm, driven by a pressure gradient [3].

Despite its distinct underlying mechanism, urinothorax often presents a diagnostic challenge. The clinical presentation can occasionally be dominated by acute respiratory symptoms, overshadowing the primary urological pathology. Furthermore, while classically described as a transudative effusion, biochemical analysis can occasionally yield discordant or exudative features, further confounding the diagnosis [4]. The gold standard for diagnosis remains a pleural fluid-to-serum creatinine ratio greater than 1.0. We present a case of a massive, rapidly reaccumulating urinothorax secondary to right-sided nephrolithiasis and hydroureteronephrosis, successfully managed with endoscopic urological intervention.

## Case presentation

A 54-year-old male with a known medical history of systemic hypertension presented to the emergency department with acute-onset, right-sided chest pain, dry cough, colicky abdominal pain, and rapidly worsening shortness of breath associated with orthopnea over the past five days. He denied any history of fever, hemoptysis, dysuria, hematuria, lower limb swelling, or recognized reduction in urine output.

On general physical examination, the patient was afebrile and hemodynamically stable, but tachypnea was prominently present. Systemic examination revealed absent breath sounds in the right infraclavicular, mammary, infra-axillary, and infrascapular regions, along with reduced vocal fremitus and vocal resonance. There was an absence of shifting dullness. Routine laboratory investigations revealed a newly diagnosed type 2 diabetes mellitus (HbA1c: 6.7%) and thrombocytosis (platelet count: 7.51 × 10⁵/µL). Renal function tests were within normal limits with a serum creatinine of 0.8 mg/dL (Table 1).

**Table 1:** Routine serological and urinalysis findings. A summary of the patient’s initial laboratory investigations upon presentation. Key findings include preserved renal function (normal serum creatinine), newly diagnosed type 2 diabetes mellitus, thrombocytosis, and microscopic hematuria without evidence of an active urinary tract infection.

Parameter	Patient’s value	Reference range	Interpretation
Blood urea nitrogen	11 mg/dL	7–20 mg/dL	Normal
Serum creatinine	0.8 mg/dL	0.7–1.3 mg/dL	Normal
Hemoglobin	9.8 g/dL	13.0–17.0 g/dL	Mild anemia
Total leukocyte count	10,060 cells/µL	4,000–11,000 cells/µL	Normal
Platelet count	8.58 lakhs/µL	1.50–4.50 lakhs/µL	Thrombocytosis
Urine protein	1+	Negative	Mild proteinuria
Urine erythrocytes	5+	Negative	Microscopic hematuria
Urine pus cells	3–5/HPF	0–5/HPF	Normal
Urine epithelial cells	2–3/HPF	0–4/HPF	Normal
Urine culture	No growth	No growth	Negative for urinary tract infection

**Figure 1:**
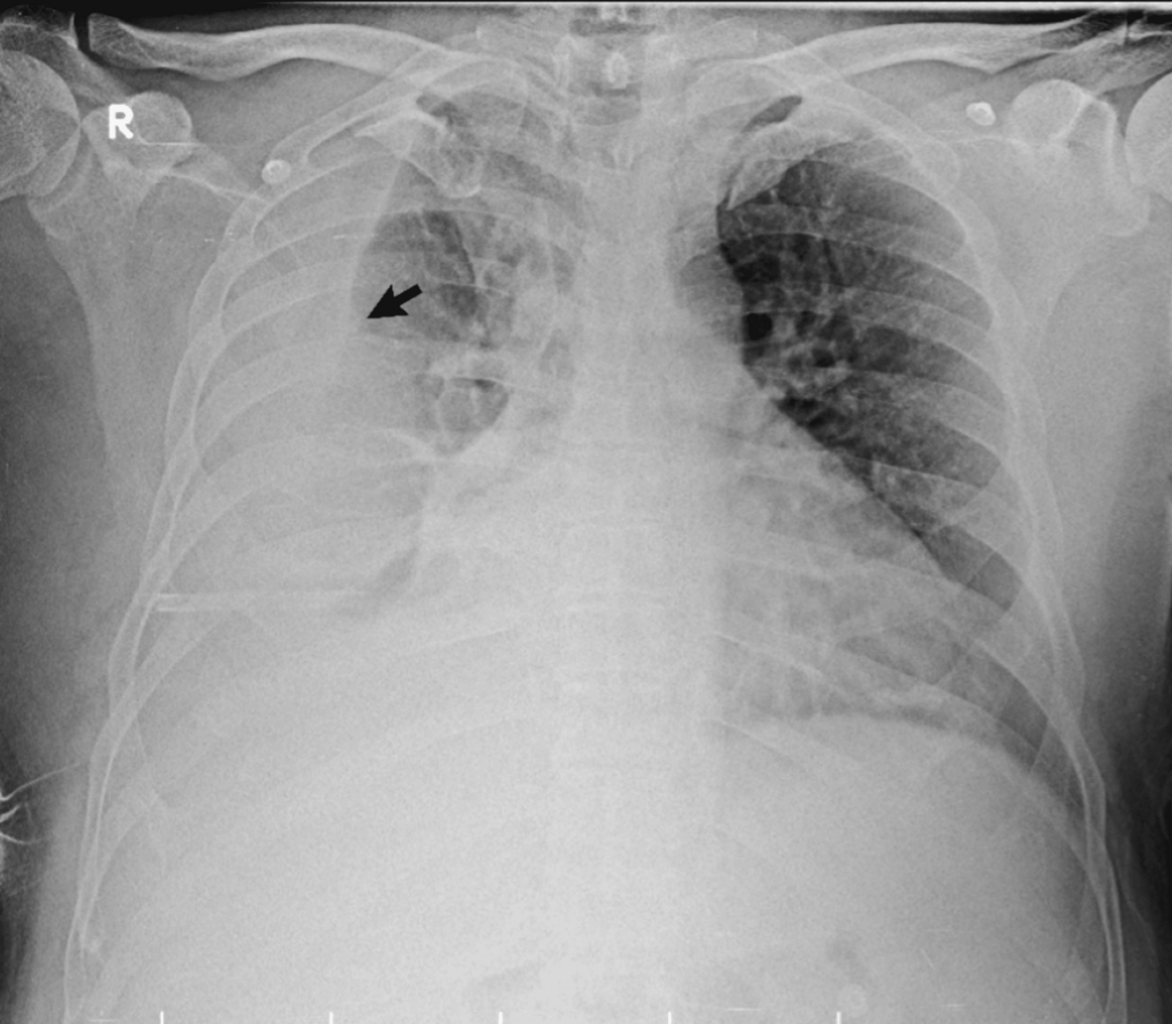
Initial chest radiograph. Anteroposterior chest radiograph obtained at presentation demonstrating a massive right-sided pleural effusion (arrow), presenting as a dense homogenous opacity that obliterates the right hemidiaphragm and costophrenic angle.

**Figure 2:**
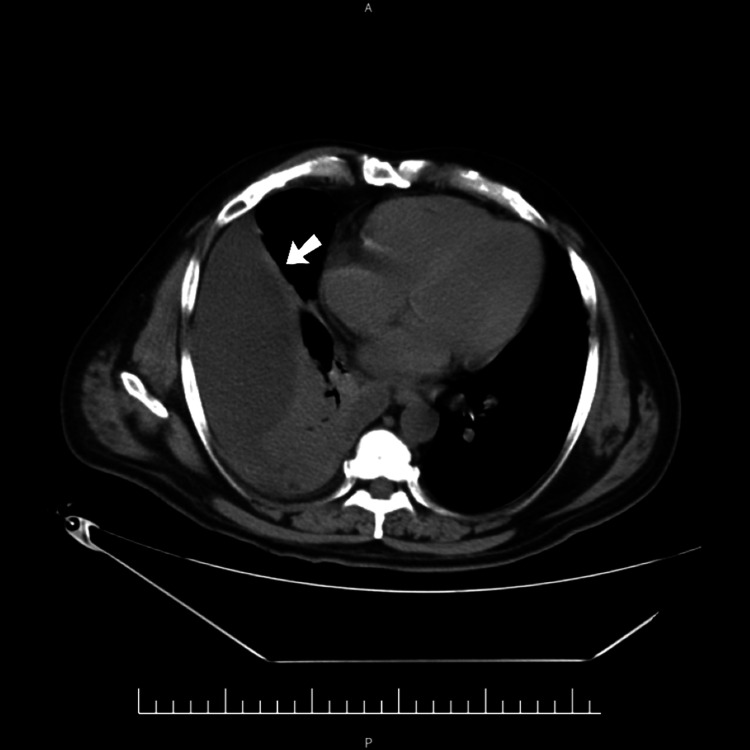
Computed tomography of the thorax. Contrast-enhanced computed tomography of the thorax (axial view) revealing a loculated, massive right pleural effusion (arrow). The fluid collection is causing complete collapse-consolidation of the right lower lobe and partial collapse of the middle and upper lobes.

**Figure 3:**
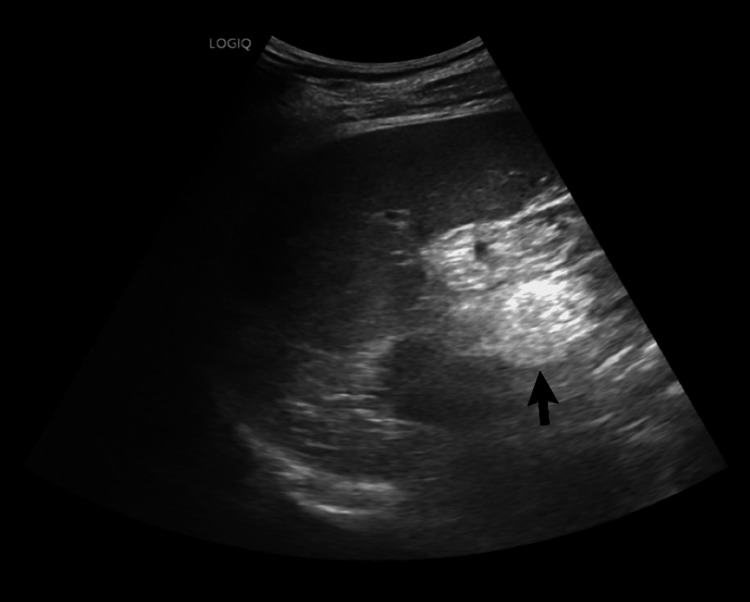
Ultrasound of the abdomen demonstrating hydroureteronephrosis. A routine grayscale ultrasound of the right upper quadrant demonstrating a dilated pelvicalyceal system (arrow) with marked parenchymal thinning, consistent with moderate-to-severe right-sided obstructive hydroureteronephrosis.

**Figure 4:**
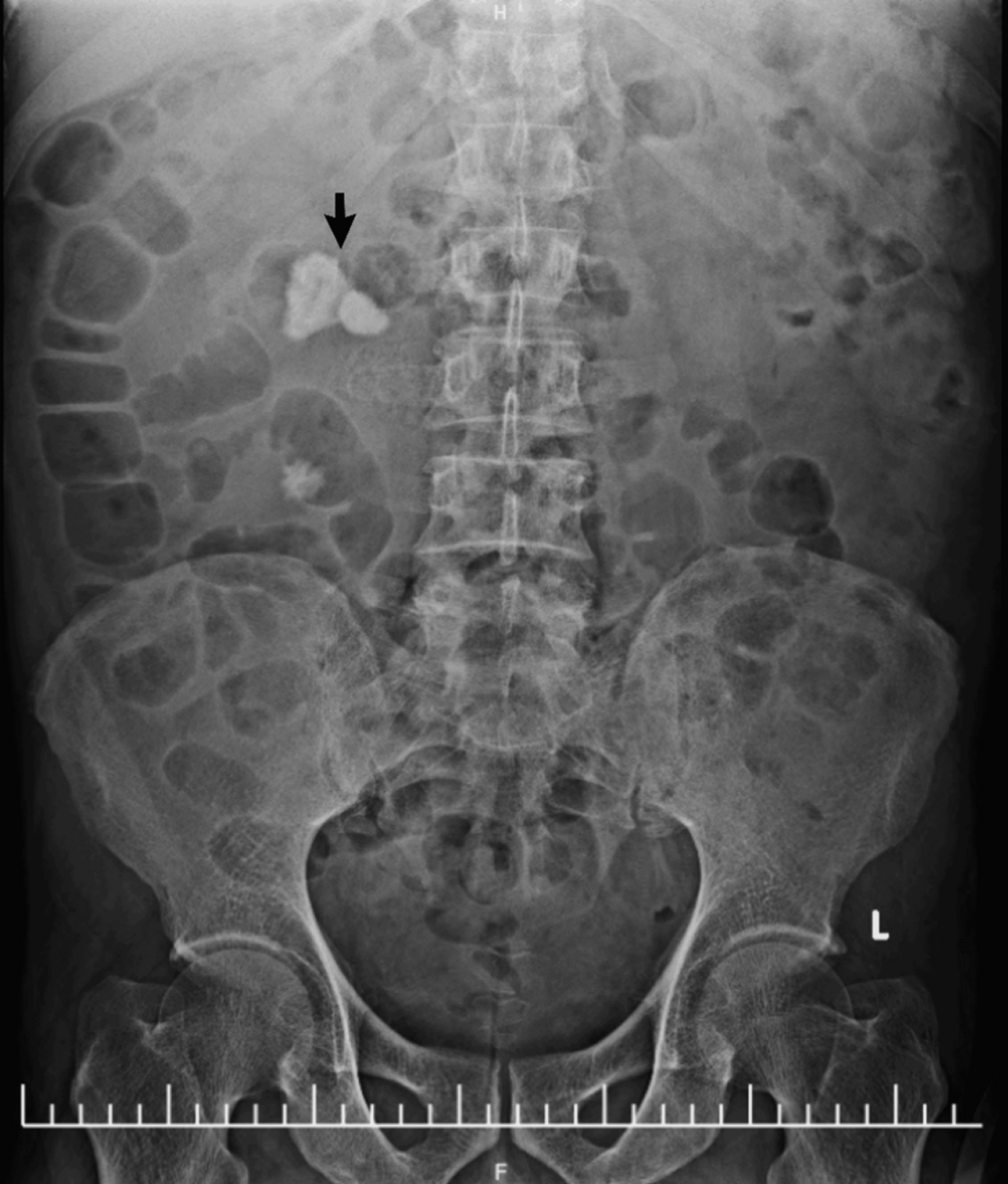
Plain radiograph of the kidneys, ureters, and bladder (KUB). Plain abdominal radiograph clearly demonstrating a large, prominent radio-opaque calculus (arrow) located in the anatomical region of the right renal pelvis and pelviureteric junction.

**Figure 5:**
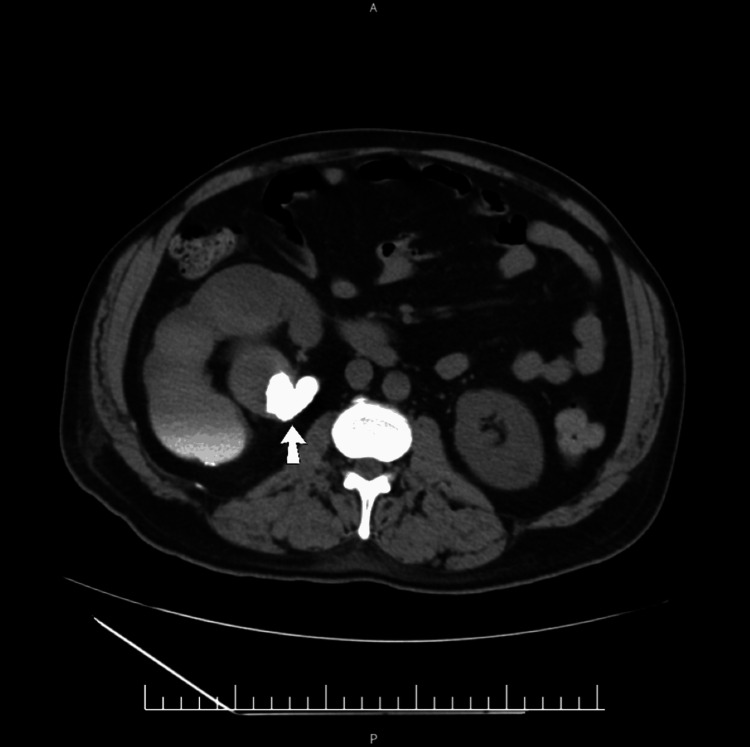
Computed tomography of the kidneys, ureters, and bladder (KUB). Contrast-enhanced computed tomography (KUB, axial view) demonstrating an enlarged right kidney with a large, highly attenuated calculus (arrow) obstructing the right pelviureteric junction. This has resulted in severe parenchymal thinning and hydronephrosis.

A chest X-ray confirmed a massive right-sided pleural effusion (Figure 1). A contrast-enhanced computed tomography (CT) of the thorax showed a massive, loculated right pleural effusion (Figure 2). A routine ultrasound of the abdomen showed obstructive moderate right hydroureteronephrosis (Figure 3). A plain radiograph (KUB) further visualized the prominent right-sided calculus (Figure 4). Subsequently, the patient proceeded with a CT of the kidneys, ureters, and bladder (KUB), which revealed a 3.3 × 2.6 × 2.3 cm calculus located at the right pelviureteric junction (PUJ) causing the hydronephrosis (Figure 5). Notably, there was no definitive radiological evidence of a retroperitoneal urinoma or frank urinary extravasation.

**Figure 6:**
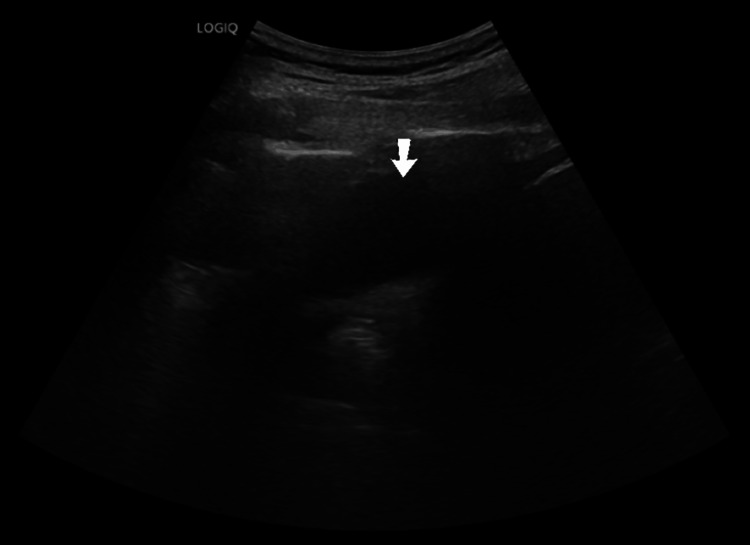
Thoracic ultrasound of right pleural effusion. Thoracic ultrasound demonstrating a large anechoic fluid collection in the right pleural space (arrow), without internal septations, corresponding to the right pleural effusion before aspiration.

**Figure 7:**
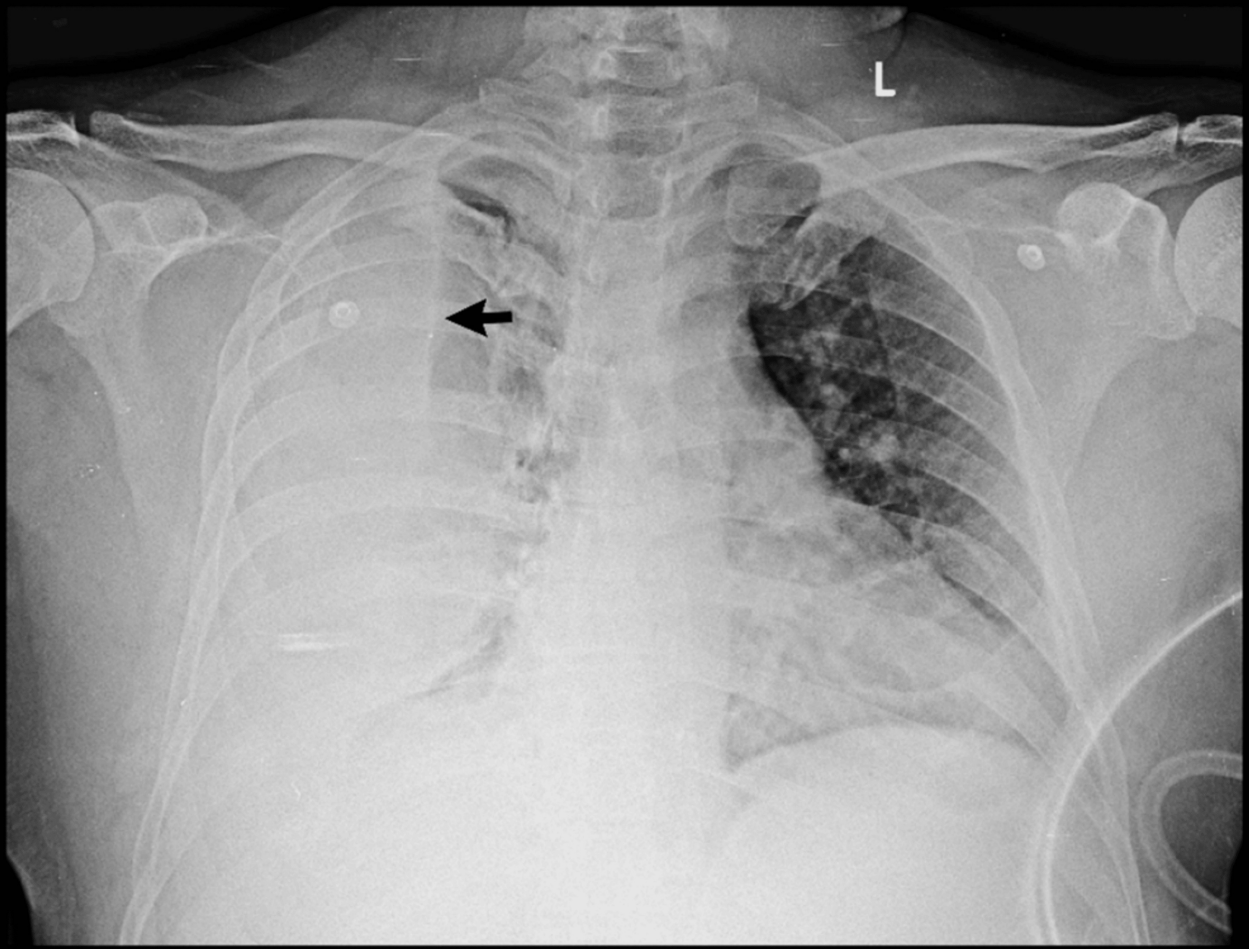
Pre-procedural chest radiograph showing effusion progression. Anteroposterior chest radiograph demonstrating progression of the massive right-sided pleural effusion (arrow) causing near-complete opacification of the right hemithorax and contralateral mediastinal shift, necessitating therapeutic thoracentesis.

**Figure 8:**
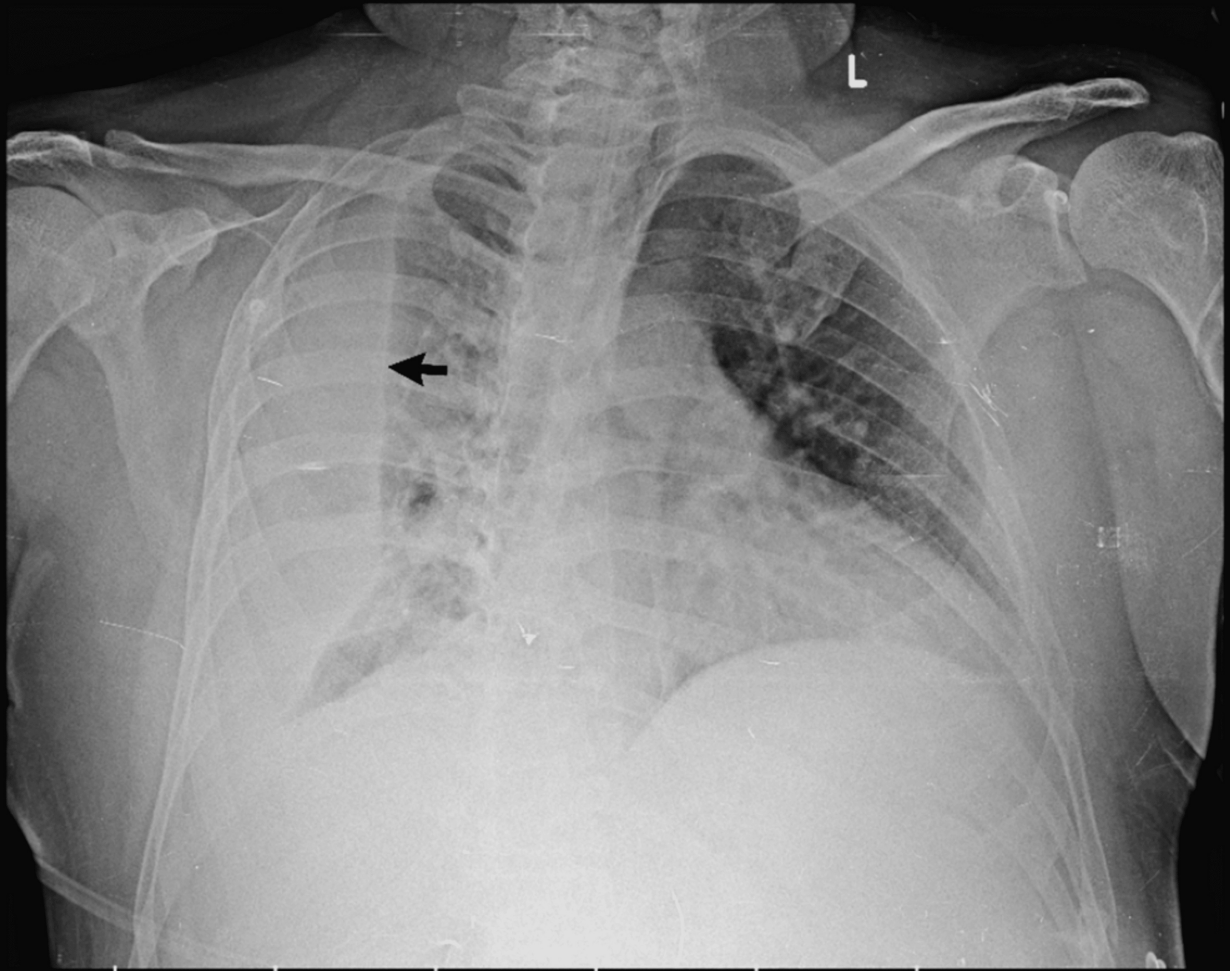
Chest radiograph post-therapeutic thoracentesis. Anteroposterior chest radiograph obtained after ultrasound-guided therapeutic thoracentesis. Despite the aspiration of fluid, there is a rapid reaccumulation and loculation of the effusion in the right mid-to-lower zones (arrow), highlighting the rapid refilling nature of the urinothorax.

To relieve the respiratory distress, ultrasound-guided therapeutic thoracentesis was initially performed (Figure 6), yielding straw-colored fluid that smelled distinctly like urine. However, the patient’s respiratory distress persisted and progressed (Figure 7). Serial chest X-rays demonstrated a rapidly refilling right pleural effusion despite aspiration (Figure 8). This necessitated repeated therapeutic pleural fluid aspirations (performed a total of five times) before any urological intervention.

Pleural fluid analysis demonstrated an adenosine deaminase of 40 U/L, lactate dehydrogenase (LDH) of 879 U/L, glucose of 80 mg/dL, and a total protein of 4.6 g/dL, consistent with an exudative profile (Table 2). The cellular differential showed polymorphonuclear leukocytes at 33.2% and mononuclear cells at 66.8%. Crucially, the pleural fluid creatinine was measured at 0.98 mg/dL. When compared to the simultaneous serum creatinine of 0.8 mg/dL, the pleural fluid-to-serum creatinine ratio was 1.22 (>1.0), confirming the diagnosis of urinothorax. Pleural fluid cultures, Genexpert MTB, and cytology were negative. Autoimmune markers (cytoplasmic antineutrophil cytoplasmic antibodies, perinuclear antineutrophil cytoplasmic antibodies, and antinuclear antibodies) were also negative.

**Table 2:** Pleural fluid biochemical and cytological analysis. Detailed analysis of the fluid obtained via therapeutic thoracentesis. The results demonstrate an atypical exudative profile with mononuclear cell predominance. Crucially, the pleural fluid creatinine was measured at 0.98 mg/dL, yielding a diagnostic pleural fluid-to-serum creatinine ratio of 1.22. Microbial cultures, acid-fast bacilli smears, and cytology were all negative.

Parameter	Patient’s value	Reference range	Interpretation
pH	8	7.60–7.64	Alkaline
Total protein	4.6 g/dL	<3.0 g/dL (transudate)	Exudative
Lactate dehydrogenase	879 U/L	<~200 U/L	Highly elevated (exudative)
Glucose	80 mg/dL	70–100 mg/dL	Normal
Adenosine deaminase	40 U/L	<40 U/L	Borderline/Normal
Total white blood cell count	334 cells/µL	<1,000 cells/µL	Low cellularity
Polymorphonuclear cells	111 cells (33.2%)	Variable	Mixed
Mononuclear cells	223 cells (66.8%)	Variable	Mononuclear predominance
Gram stain and AFB smear	Occasional pus cells; no organisms or AFB seen	Negative	Negative for acute bacterial/tubercular infection
Culture	No growth	No growth	Sterile
Cytology	Negative for malignant cells	Negative	No evidence of malignancy

**Figure 9:**
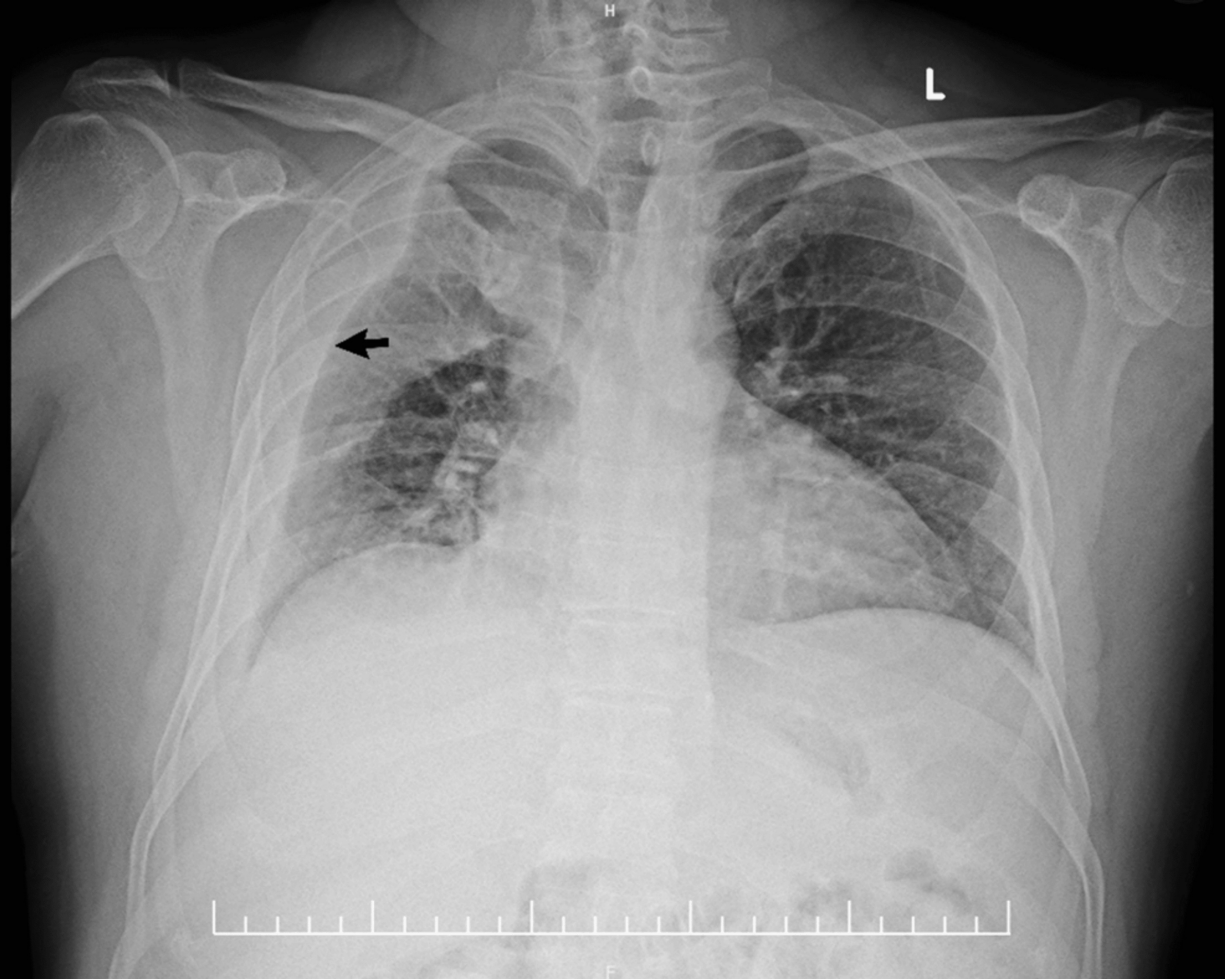
Postoperative chest radiograph demonstrating resolution. Posteroanterior chest radiograph taken after the patient underwent right percutaneous nephrolithotomy and double-J stenting. The image demonstrates near-complete resolution of the right-sided urinothorax and good expansion of the underlying lung parenchyma, confirming the successful definitive treatment.

The patient was managed with a multidisciplinary approach. To definitively address the underlying urological pathology, he underwent right percutaneous nephrolithotomy (PCNL) and double-J (DJ) stenting under general anesthesia. Post-procedure, the patient was clinically better, and a repeat chest X-ray showed near-complete resolution of the effusion with good lung expansion (Figure 9). He was subsequently discharged in stable condition on room air.

## Discussion

Urinothorax is a rare pleural effusion of extravascular origin that requires a high index of suspicion, particularly when a patient’s acute respiratory distress completely overshadows their underlying urological symptoms. The condition primarily affects adults and is most often ipsilateral to the obstructed urinary tract, though bilateral cases have been reported [1,2]. The pathophysiology hinges on the accumulation of perirenal urine following an obstruction, such as the large right PUJ calculus seen in our patient. This retroperitoneal fluid follows the path of least resistance, tracking cephalad and crossing the diaphragm into the pleural space via lymphatic channels or anatomical micro-defects [3]. Classically, a retroperitoneal urinoma is considered the necessary pathophysiological intermediate for a urinothorax. However, as demonstrated in our case, urinomas are frequently radiologically occult. The urological and radiological literature notes that this absence of classic radiological findings is not uncommon and can significantly contribute to diagnostic delays [3,4].

While Light’s criteria generally classify a urinothorax as a transudate (characterized by low protein and low pH of <7.30), recent literature has increasingly documented atypical or discordant biochemical profiles [4,5]. Our case reinforces this evolving understanding, as the patient presented with a distinctly exudative fluid profile featuring an elevated LDH and total protein. The lack of typical findings, specifically the exudative nature of the fluid and the absence of a radiologically demonstrable urinoma or anatomical connection, does not rule out a urinothorax. Instead, the diagnosis in our patient was strongly supported by a distinct constellation of clinical features: the characteristic uriniferous odor of the aspirate, the rapid reaccumulation of pleural fluid requiring five therapeutic aspirations before intervention, and, most crucially, the complete clinical and radiological resolution of the effusion following PCNL and DJ stenting.

Clinical synthesis and pattern recognition

To aid in differential diagnosis and pattern recognition, clinicians must remain vigilant for these atypical presentations. When evaluating a patient with an unexplained exudative effusion, the differential diagnosis typically prioritizes infectious etiologies (such as empyema or tuberculosis) and malignancy. However, the presence of a rapidly accumulating, recurrent pleural effusion in the known or suspected setting of acute obstructive uropathy (such as hydronephrosis) is a critical pattern recognition clue.

In such overlapping scenarios, calculating the pleural fluid-to-serum creatinine ratio is the definitive step. Because creatinine is a large molecule that does not readily cross the pleural membrane under normal conditions, a pleural fluid concentration that exceeds the serum concentration (a ratio >1.0) is considered pathognomonic [1,4]. In our patient, a raised diagnostic creatinine ratio of 1.22 served as the ultimate differentiator. This metric decisively separated the urinothorax from cardiopulmonary or infectious etiologies, circumvented the confusing exudative fluid profile, and directly guided the need for definitive, curative urological intervention.

## Conclusions

This case highlights that urinothorax should remain on the differential diagnosis for patients presenting with an unexplained pleural effusion and concurrent obstructive uropathy, even when classical radiological or biochemical features are absent. While traditionally described as a transudate, our findings suggest that atypical, exudative profiles can occur, likely secondary to localized pleural inflammation. It is important to note that, as a single case report limited by the absence of advanced anatomical imaging (such as magnetic resonance imaging) to definitively trace a fistulous tract, these observations cannot be broadly generalized. Nevertheless, this case underscores the diagnostic utility of the pleural fluid-to-serum creatinine ratio. A ratio greater than 1.0 remains a highly reliable marker that can circumvent confusing fluid profiles and appropriately guide definitive urological intervention.
